# Beyond the Usual Suspects: Appendiceal Bleeding as the Surprising Cause of Lower Gastrointestinal (GI) Bleeding

**DOI:** 10.7759/cureus.76663

**Published:** 2024-12-31

**Authors:** Asher Siddiqui, Nowera Zafar, Mahdi Hakiminezhad, Zohaib Jamal, Imran Alam, Zeeshan Khawaja

**Affiliations:** 1 Department of Surgery, Wrightington, Wigan and Leigh NHS Foundation Trust, Wigan, GBR; 2 Department of Radiology, Mersey and West Lancashire Teaching Hospitals NHS Foundation Trust, Lancaster, GBR

**Keywords:** appendicular bleed, ct angiogram, laparoscopic appendicectomy, lower gi bleed, the appendix

## Abstract

Lower gastrointestinal bleeding (LGIB) is a common clinical condition typically associated with diseases like diverticular disease, inflammatory bowel disease, and cancer. However, rarer etiologies such as appendiceal hemorrhage can present similarly, complicating diagnosis and management. This case report discusses a 42-year-old male who presented with severe rectal bleeding. Despite a comprehensive diagnostic workup, including colonoscopy and CT angiography, the underlying cause was identified as an obscure appendiceal hemorrhage.

This atypical presentation underscores the importance of considering a broad differential diagnosis, even in cases with seemingly straightforward clinical features. While laparoscopic appendectomy is the standard surgical approach for managing appendiceal hemorrhage, a conservative management strategy was successfully employed in this particular case, highlighting the importance of individualized patient care and judicious clinical decision-making.

This case report emphasizes the necessity of a multidisciplinary approach, involving careful clinical assessment, advanced imaging, and endoscopic evaluation, to accurately diagnose and manage complex cases of gastrointestinal bleeding. By recognizing and addressing uncommon etiologies, clinicians can improve patient outcomes and minimize unnecessary interventions.

## Introduction

Lower gastrointestinal bleeding (LGIB), a potentially life-threatening condition, is defined as hemorrhage originating from the GI tract below the ligament of Treitz [1]. LGIB is most often linked to conditions affecting the colon, rectum, and terminal ileum [2]. Despite advancements in radiological and colonoscopic techniques, accurately diagnosing LGIB, especially in cases of severe or intermittent bleeding, remains a significant clinical challenge. Despite the utilization of various diagnostic modalities, including angiography, multidetector computed tomography (MDCT), and colonoscopy, the precise identification of the bleeding source remains elusive in approximately 10% of cases [3-5]. Typical etiologies of LGIB include vascular disease, Crohn's disease, neoplasia, inflammation, hemorrhoids, and ischemic colitis [6].

Appendiceal hemorrhage as a cause of LGIB is an uncommon occurrence, with only a few isolated case reports documented in the literature. Moreover, appendiceal bleeding itself is an exceedingly rare event, accounting for a mere 0.014% of all GI bleeding cases [7]. Laparoscopic appendectomy is considered the standard of care for appendiceal hemorrhage [8].

This case report details a perplexing instance of severe rectal bleeding in a young male patient. Despite a comprehensive diagnostic workup, the underlying etiology was ultimately identified as an obscure appendiceal hemorrhage. This unusual presentation underscores the diagnostic challenges associated with obscure GI bleeding, even in the era of advanced imaging techniques. While laparoscopic appendectomy is the standard surgical approach for managing appendiceal hemorrhage, a conservative management strategy was successfully employed in this particular case, highlighting the importance of individualized patient care and judicious clinical decision-making. This report underscores the importance of considering uncommon etiologies, even in cases of seemingly straightforward presentations of GI bleeding.

## Case presentation

A 42-year-old male with a past medical history of myocardial infarction, type 2 diabetes mellitus, and hypertension presented with a one-week history of melena. Initially attributed to a known history of hemorrhoidal bleeding, the patient presented with a change in stool color to black and a foul odor, suggesting a more significant gastrointestinal bleed. Two days before presentation, he sought medical attention and underwent blood testing by his general practitioner. Subsequent laboratory results revealed a significant drop in hemoglobin levels from 142 g/L to 65 g/L, prompting urgent hospital admission.

The patient's clinical presentation in hospital included orthostatic dizziness, exertional dyspnea, and fatigue. He denied experiencing chest pain, palpitations, syncope, loss of appetite, or weight loss. Furthermore, he reported no recent changes in bowel habits and no use of over-the-counter medications, non-steroidal anti-inflammatory drugs, or herbal supplements. His social history revealed a history of smoking cessation and occasional alcohol consumption.

On physical examination, the patient was alert and receiving a blood transfusion. Cardiovascular examination revealed normal heart sounds, and respiratory examination was clear. Abdominal examination was unremarkable, with a soft, non-tender abdomen. There was no evidence of peripheral edema. Vital signs were stable, with a normal temperature, pulse rate, blood pressure, respiratory rate, and oxygen saturation.

Laboratory investigations revealed significant anemia. A detailed summary of the patient's blood test results, including initial values and trends observed during hospitalization, is presented in Table 1. Iron studies are summarized in Table 2.

**Table 1:** Serial blood test results: admission to discharge. Hb, hemoglobin; RBC, red blood cells; MCV, mean corpuscular volume; MCH, mean corpuscular hemoglobin; WBC, white blood cell; eGFR, estimated glomerular filtration rate; AKI, acute kidney injury; ALP, alkaline phosphatase; ALT, alanine transaminase

Blood test parameters	Admission day	Trend during the admission					Reference values
	Day 0	Day 1	Day 2	Day 3	Day 4	Day 5	
Full blood count							
Hb	65	60	86	90	85	90	130-180 g/L
RBC	2.15	1.98	2.83	2.93	2.77	2.99	4.50-5.50 x 10^12^/L
Hematocrit	0.19	0.17	0.24	0.25	0.24	0.27	0.40-0.50 L/L
MCV	89	87	86	87	87	90	80-100 fL
MCH	30.2	30.3	30.4	30.7	30.7	30.1	27-32 pg
WBC	11.9	8.4	10.1	9	6.6	5.7	4-11 x 10^9^/L
Platelets	304	219	191	207	230	311	130-450 x 10^9^/L
Renal function tests							
Urea	6.8	6.9	4.8	6.2		2.5	2.5-7.8 mmol/L
Creatinine	68	62	63	142		58	65-104 umol/L
eGFR	>90	>90	>90	>90		>90	>90 mL/minute/1.73 m^2^
AKI score	0	0	0	0		0	0-0
Serum electrolytes							
Sodium	140	140		142		139	133-146 mmol/L
Potassium	3.8	4		3.7		3.8	3.5-5.3 mmol/L
Phosphate	1.14		1.06			1.14	0.80-1.50 mmol/L
Magnesium	0.68	0.68		0.76		0.73	0.70-1 mmol/L
Albumin	38	29	33	33	33	34	35-50 g/L
Liver function tests							
Total bilirubin	4	10	17	6	17	6	0-20 umol/L
ALP	73	44	41	65	41	65	30-130 U/L
ALT	22	16	19	29	19	29	7-40 U/L

**Table 2:** Iron studies results on day 1 and day 5 of admission.

Iron studies parameters	Day 1	Day 5	Reference values
Ferritin	4		22-322 ug/l
Transferrin	3	2.52	2.15-3.65 g/L
Serum iron	2.1	2.5	11-31 umol/L
Iron saturation	3	4	15%-45%

**Figure 1:**
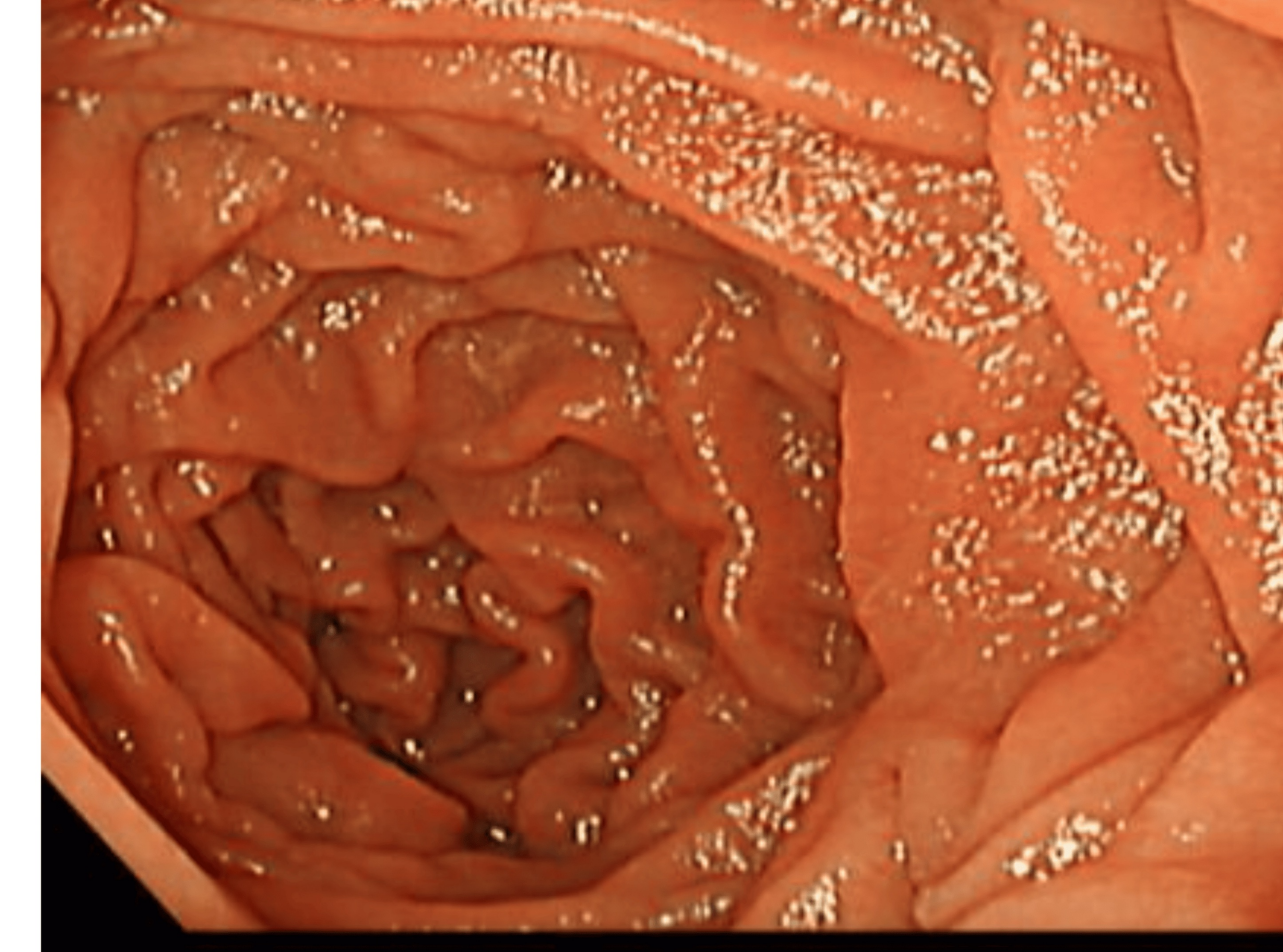
EGD visualization of the upper gastrointestinal tract, showing no signs of inflammation, erosion, or bleeding. EGD,  esophagogastroduodenoscopy

The patient was managed conservatively and received two units of packed red blood cells. An upper esophagogastroduodenoscopy (EGD) revealed minimal erosions in the gastric antrum but no active bleeding or ulceration (Figure 1). A biopsy for Clostridium difficile toxin was taken.

**Figure 2:**
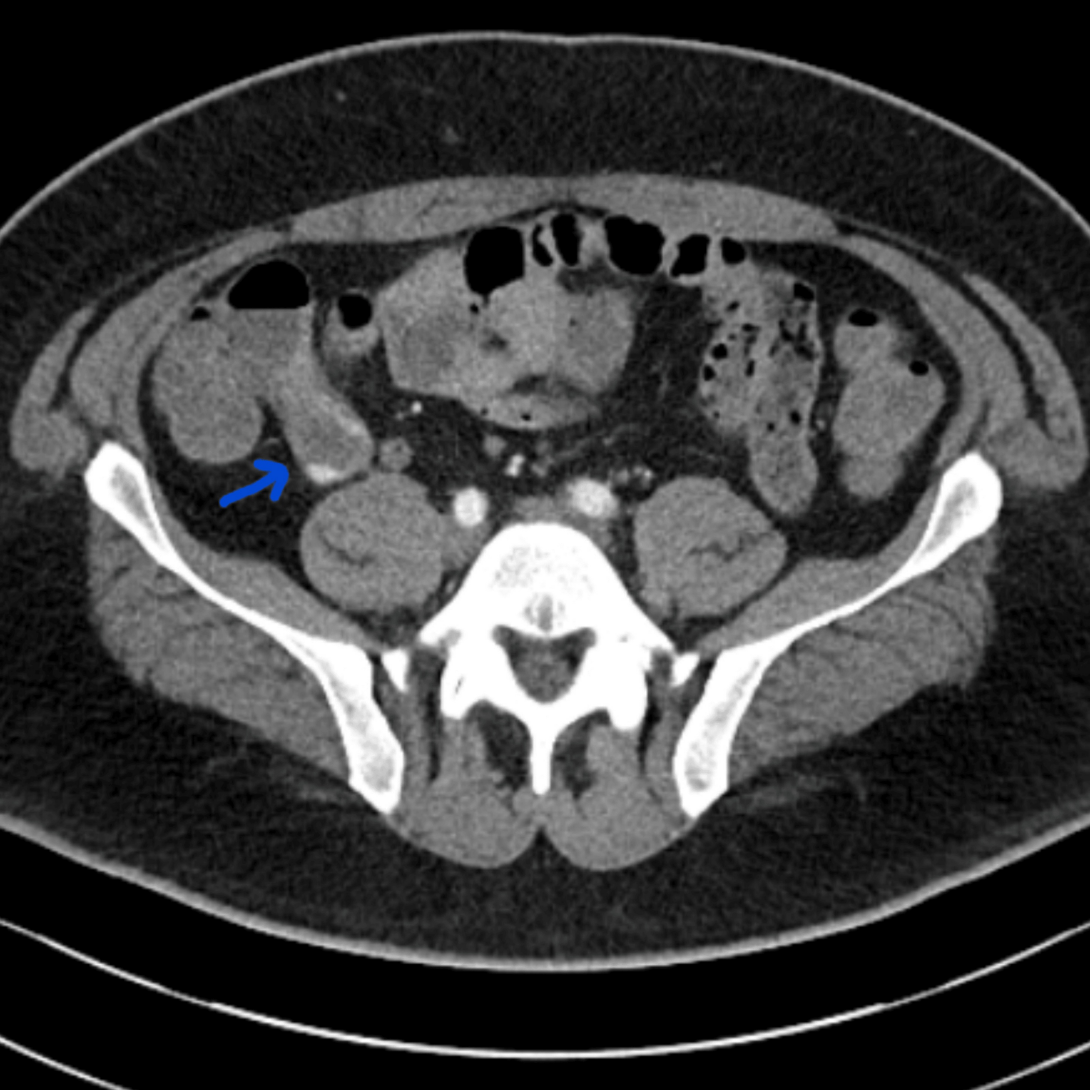
Arterial-phase CT angiogram showing focal contrast extravasation within the thickened appendix, indicative of active bleeding. CT, computed tomography

**Figure 3:**
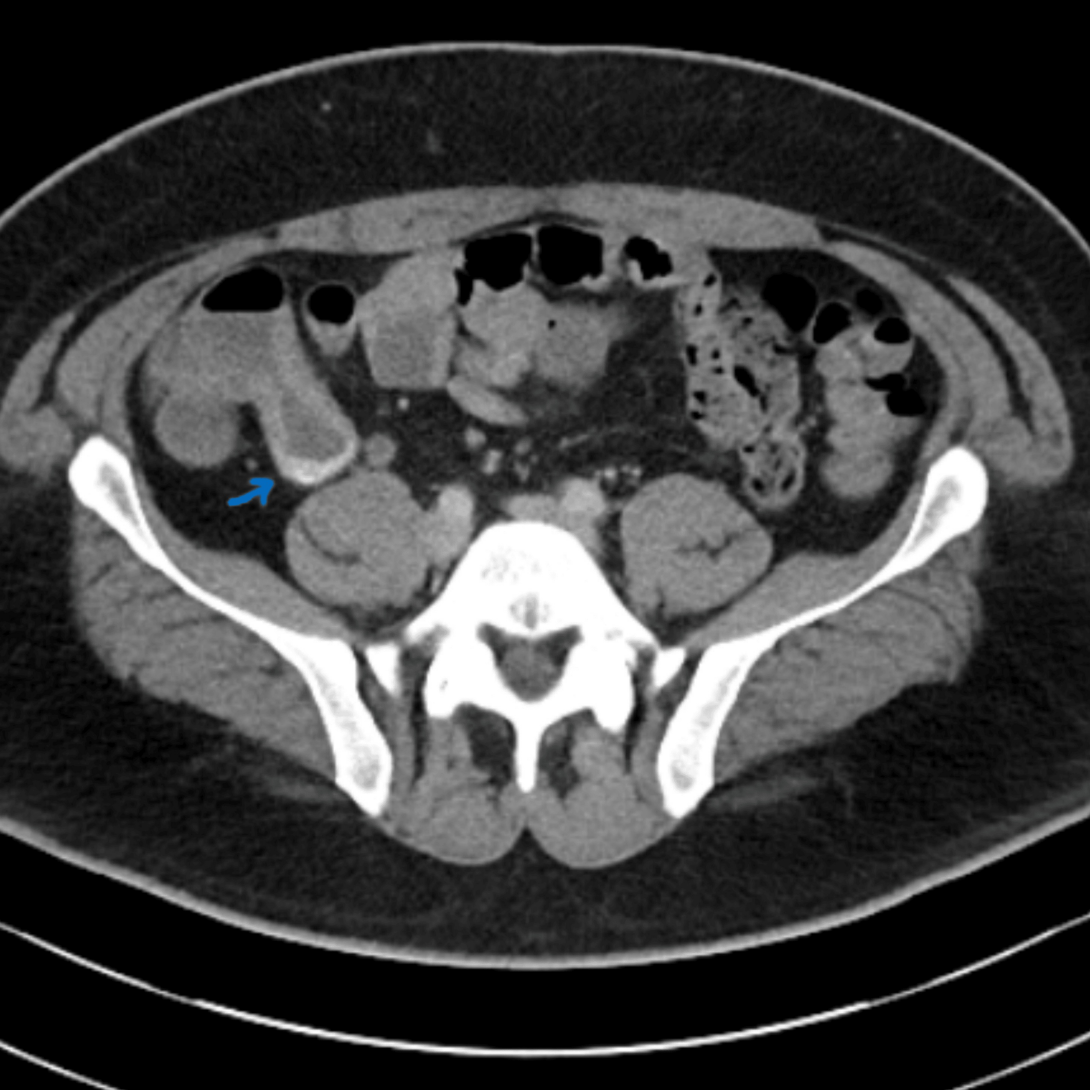
Venous-phase CT angiogram showing prominent contrast extravasation within the thickened appendix, indicative of active bleeding. CT, computed tomography

The following day, the patient experienced a recurrent episode of fresh rectal bleeding and melena. Despite receiving two units of packed red blood cells, a repeat hemoglobin level revealed a further decline to 62 g/L. Following a discussion with the surgical team, the patient underwent an urgent CT angiography (CTA) of the abdomen and pelvis. The CTA revealed a thickened appendix measuring approximately 17 mm in diameter at its proximal end, with a normal-sized terminal segment and no significant surrounding inflammatory changes. A focal area of contrast extravasation was identified in the arterial phase, which appeared to progress toward the dependent portion of the appendix in the portal venous phase, suggestive of active intra-luminal bleeding (Figures 2-3).

**Figure 4:**
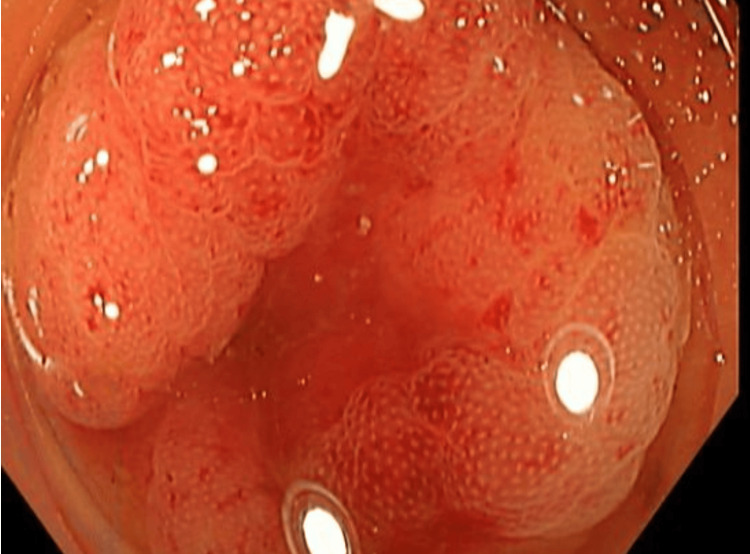
Colonoscopy image of the appendix demonstrating normal mucosal appearance without evidence of active bleeding.

**Figure 5:**
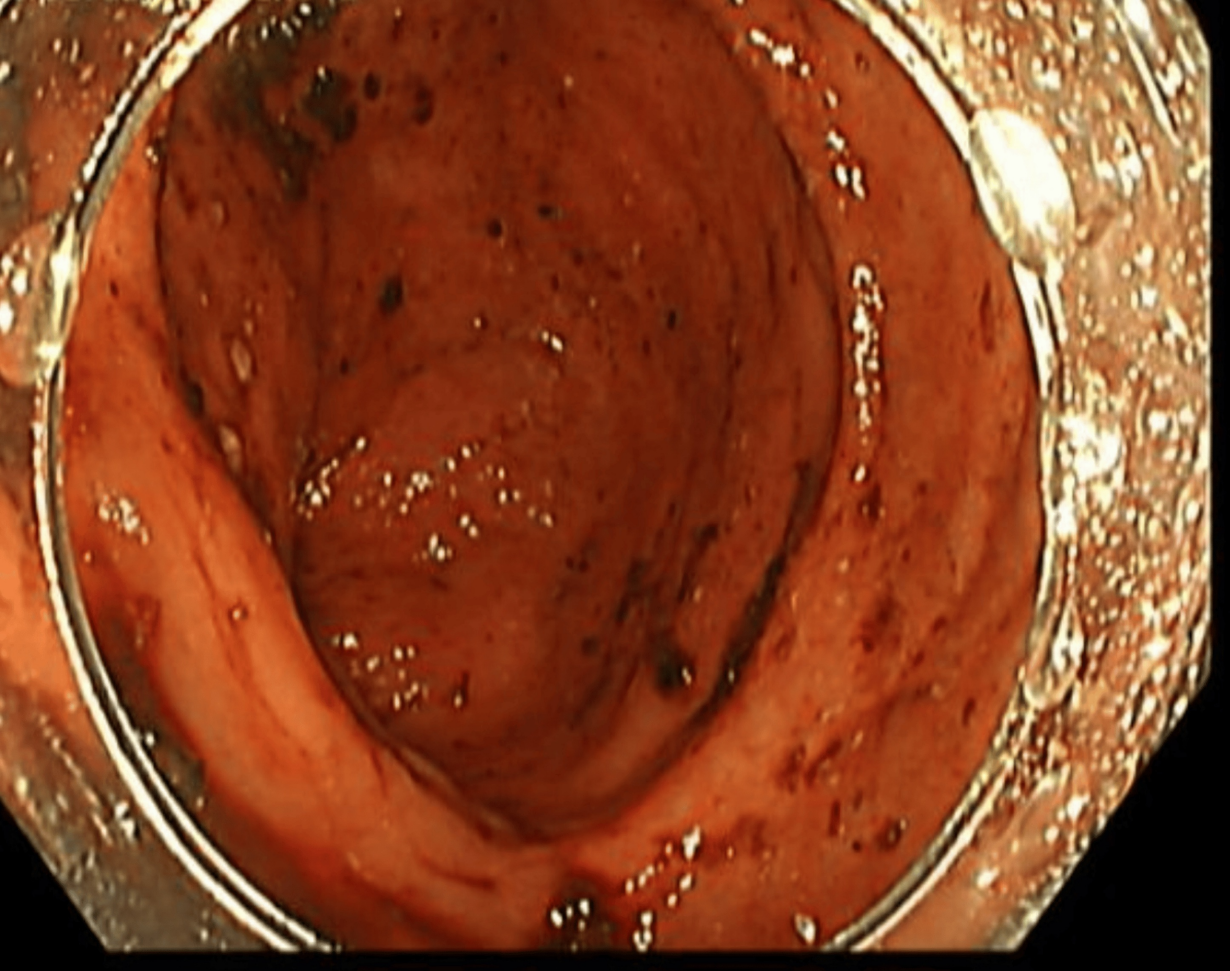
Colonoscopy image of the cecum demonstrating scattered areas of old blood on the mucosal surface.

**Figure 6:**
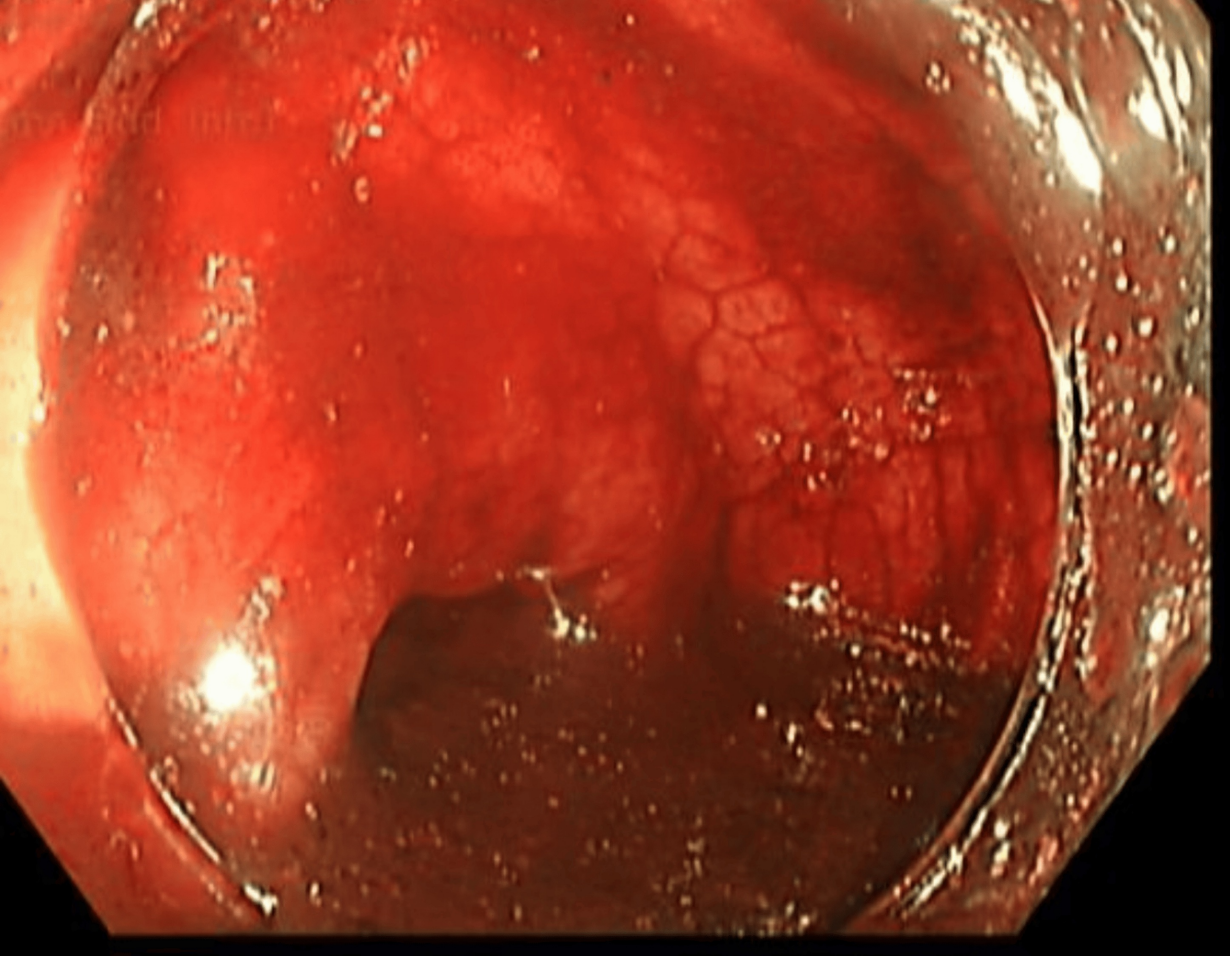
Colonoscopy image of the transverse colon demonstrating scattered areas of old blood on the mucosal surface.

**Figure 7:**
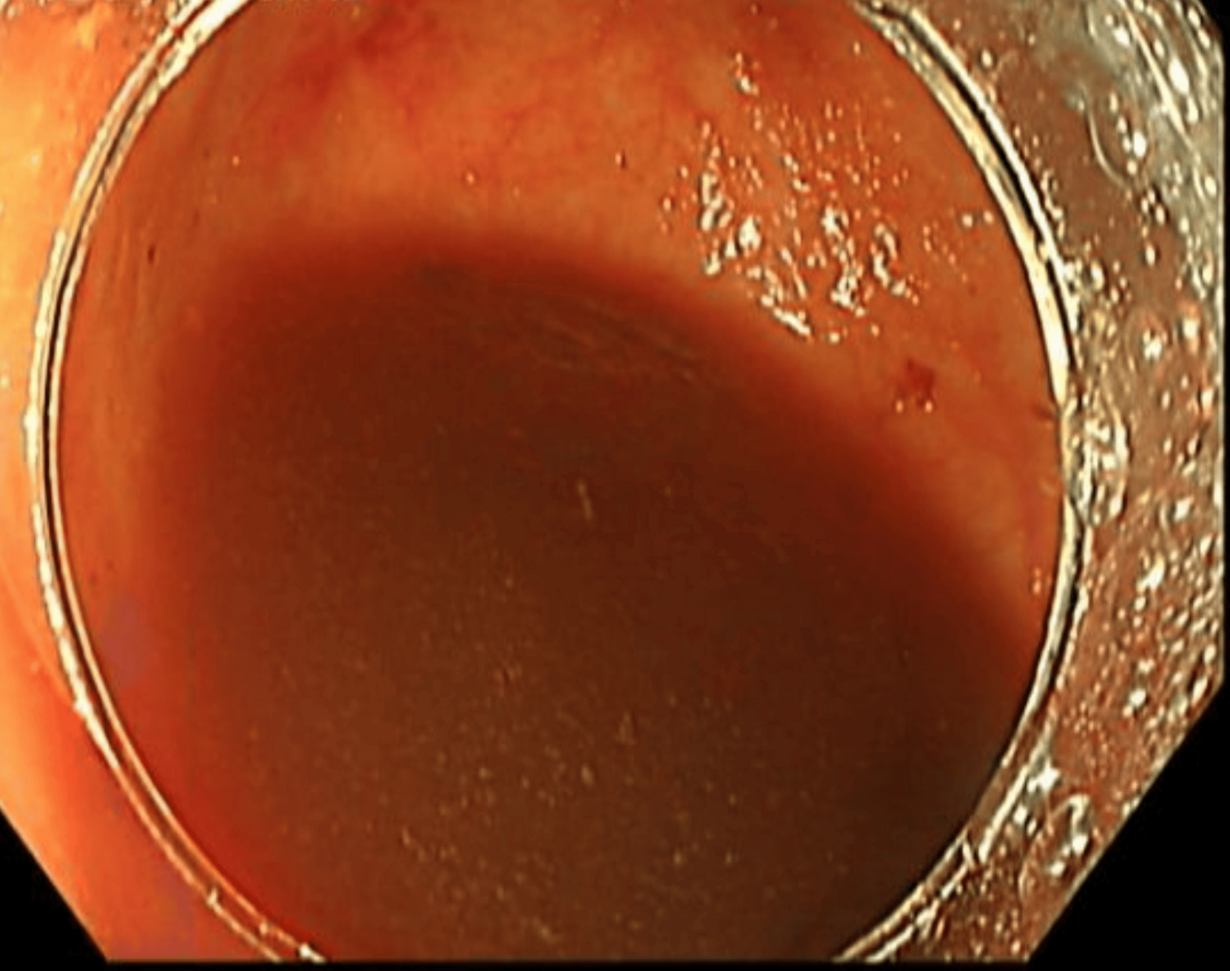
Colonoscopy image of the descending colon demonstrating pooling of old blood on the mucosal surface.

Initially, a laparoscopic appendectomy was planned. However, after further discussion with a multidisciplinary team involving a gastroenterologist, radiologist, colorectal surgeon, and general surgeon, a colonoscopy was performed. The colonoscopy revealed a large volume of old blood present throughout the colon, significantly limiting visualization of the colonic mucosa. Despite this limitation, no active bleeding source was identified within the colon or appendix (Figures 4-7). The cecum and appendix were closely monitored specifically for 10 minutes during the procedure to ensure no evidence of active bleeding.

Following the colonoscopy, which excluded active bleeding sources, a conservative management approach was adopted. Close monitoring was implemented, with a plan for a laparoscopic appendectomy if further bleeding occurred. During this hospitalization, the patient received a total of eight units of packed red blood cells. After a six-day observation period without recurrent bleeding, the patient was discharged. He was advised to seek immediate medical attention for any further bleeding episodes. A follow-up appointment was scheduled with his general practitioner for a repeat blood test to monitor hemoglobin levels and exclude occult bleeding.

## Discussion

LGIB is a frequent presentation in the emergency, accounting for 3% of emergency surgical referrals [1]. The differential diagnosis for LGIB is extensive and typically includes inflammatory bowel disease, diverticulitis, ischemic colitis, cancer, hemorrhoids, and occasionally arteriovenous malformations [6]. Appendiceal hemorrhage as the cause of LGIB is a rare event, with a limited number of cases reported in the literature, most of which involve middle-aged male patients. The most common presenting clinical symptom is hematochezia [9].

Multi-phase abdominal CT imaging and colonoscopy have revolutionized medicine with the introduction of non-invasive to minimally invasive methods to enhance diagnostic accuracy [4,10]. In our described case as well, the CT scan was pivotal in establishing the diagnosis, as the arterial phase showed blush of contrast at the appendix. However, despite advances in radiology and gastroenterology, pinpointing the exact source of bleeding can be challenging, which complicates management. The colonoscopy performed in this case was instrumental in ruling out active hemorrhage in the large intestine, thereby facilitating a decision in favor of conservative treatment.

The choice of treatment is generally dependent on the patient's clinical and frailty status and the etiology of the bleed. The etiologies of appendiceal bleeding discussed in the literature include benign erosions and ulcerations, appendiceal angiodysplasia, appendicitis (ulcerative, granulomatous), appendectomy-associated bleeding, carcinomas, and Dieulafoy lesions of the appendix [11]. Consequently, treatment modalities for appendiceal bleeding have largely included surgical intervention (laparoscopic appendectomy), with occasional use of angiography coil intervention and hemostatic clipping [7, 10-11]. Although hemostasis through endoscopy is a viable option in LGIB cases, no case detailing the exclusive use of this modality has been published for appendiceal hemorrhage in particular. 

In our case, an exclusively conservative treatment modality for appendiceal hemorrhage was employed. A conservative approach is discussed in one of the cases from the case series studied at the West China Hospital of Sichuan University where no treatment was used, and there was no recurrent bleeding at the four-month follow-up [7]. However, in that case, the patient despite being symptomatic and having positive CT and colonoscopy findings, did not have a drop in his hemoglobin. Another case discussed in the same paper also mentions use of non-surgical options with no re-bleeding at the five-month follow-up [7].

Recognizing the possibility of this rare diagnosis as a potential cause for patient's symptoms of LGIB is crucial to the role of a clinician. Overall, our case and the literature suggest that the diagnosis of appendiceal bleeding is challenging; however, on the positive side, the prognosis is generally good. Multiphase CT imaging and colonoscopy are crucial in establishing the diagnosis. Primarly, appendectomy is preferred. However, the exact therapeutic choice has to be tailored as per the specific patient.

## Conclusions

In conclusion, this case report illustrates the diagnostic challenges encountered in obscure LGIB, despite advancements in imaging. While laparoscopic appendectomy is the standard surgical approach for appendiceal hemorrhage, this case demonstrates the successful application of a conservative management strategy. This highlights the importance of individualized patient care and emphasizes the need to consider uncommon etiologies like appendiceal hemorrhage in the differential diagnosis of LGIB. A multidisciplinary approach, encompassing careful clinical assessment, advanced imaging, and endoscopic evaluation, is crucial for navigating the diagnostic and therapeutic challenges associated with such cases.
